# Comparative genomics of *Brachyspira pilosicoli* strains: genome rearrangements, reductions and correlation of genetic compliment with phenotypic diversity

**DOI:** 10.1186/1471-2164-13-454

**Published:** 2012-09-05

**Authors:** Luke J Mappley, Michael L Black, Manal AbuOun, Alistair C Darby, Martin J Woodward, Julian Parkhill, A Keith Turner, Matthew I Bellgard, Tom La, Nyree D Phillips, Roberto M La Ragione, David J Hampson

**Affiliations:** 1 Department of Bacteriology, Animal Health and Veterinary Laboratories Agency, Reading University, Addlestone, Surrey, KT15 3NB, UK; 2 Department of Food and Nutritional Sciences, University of Reading, Reading, Berkshire, RG6 6AP, UK; 3 School of Medical Sciences, Edith Cowan University, Perth, WA, 6027, Australia; 4 Institute of Integrative Biology, School of Biological Sciences, University of Liverpool, Liverpool, L69 7ZB, UK; 5 The Wellcome Trust Sanger Institute, Genome Campus, Hinxton, Cambridge, CB10 1SA, UK; 6 Discuva Ltd, Cambridge Science Park, Milton Road, Cambridge, CB4 0WE, UK; 7 School of Veterinary and Biomedical Science, Murdoch University, Perth, WA, 6150, Australia; 8 Faculty of Health and Medical Sciences, University of Surrey, Guilford, Surrey, GU2 7XH, UK

**Keywords:** *Brachyspira pilosicoli*, Spirochaete, Colitis, Zoonosis, Whole genome sequencing, Genome comparison, Horizontal gene transfer, Phenotype MicroArray™

## Abstract

**Background:**

The anaerobic spirochaete *Brachyspira pilosicoli* causes enteric disease in avian, porcine and human hosts, amongst others. To date, the only available genome sequence of *B. pilosicoli* is that of strain 95/1000, a porcine isolate. In the first intra-species genome comparison within the *Brachyspira* genus, we report the whole genome sequence of *B. pilosicoli* B2904, an avian isolate, the incomplete genome sequence of *B. pilosicoli* WesB, a human isolate, and the comparisons with *B. pilosicoli* 95/1000. We also draw on incomplete genome sequences from three other *Brachyspira* species. Finally we report the first application of the high-throughput Biolog phenotype screening tool on the *B. pilosicoli* strains for detailed comparisons between genotype and phenotype.

**Results:**

Feature and sequence genome comparisons revealed a high degree of similarity between the three *B. pilosicoli* strains, although the genomes of B2904 and WesB were larger than that of 95/1000 (~2,765, 2.890 and 2.596 Mb, respectively). Genome rearrangements were observed which correlated largely with the positions of mobile genetic elements. Through comparison of the B2904 and WesB genomes with the 95/1000 genome, features that we propose are non-essential due to their absence from 95/1000 include a peptidase, glycine reductase complex components and transposases. Novel bacteriophages were detected in the newly-sequenced genomes, which appeared to have involvement in intra- and inter-species horizontal gene transfer. Phenotypic differences predicted from genome analysis, such as the lack of genes for glucuronate catabolism in 95/1000, were confirmed by phenotyping.

**Conclusions:**

The availability of multiple *B. pilosicoli* genome sequences has allowed us to demonstrate the substantial genomic variation that exists between these strains, and provides an insight into genetic events that are shaping the species. In addition, phenotype screening allowed determination of how genotypic differences translated to phenotype. Further application of such comparisons will improve understanding of the metabolic capabilities of *Brachyspira* species.

## Background

Spirochaetes represent a monophyletic lineage and a major branch in eubacterial evolution; *Brachyspira* is the sole genus of the family *Brachyspiraceae* within the order Spirochaetales, which belongs to the spirochaete phylum [1]. *Brachyspira* are Gram-negative, loosely coiled, aerotolerant anaerobes that colonise the lower gastrointestinal (GI) tract of mammals and birds, but vary in pathogenicity. There are seven *Brachyspira* species that are currently officially recognised: *B. aalborgi*, a potential human pathogen [2]; the porcine pathogen, *B. hyodysenteriae*[3]; the avian pathogens, *B. alvinipulli*[4] and *B. intermedia*[5]; the avian, porcine and human pathogen, *B. pilosicoli*[6]; non-pathogenic *B. innocens*[7] and *B. murdochii*, which is of uncertain pathogenic potential [5]. In addition, there are a number of proposed species including “*B. canis*” [8], “*B. pulli*” [9] and “*B. suanatina*” [10] amongst others. The classification of the genus is still immature and the often used descriptors of certain *Brachyspira* as pathogenic, intermediate pathogenic or non-pathogenic is subject to debate.

*B. pilosicoli* is the only species considered to be a pathogen of birds, pigs and humans. The species is quite diverse, and it seems unlikely that there are barriers to cross-species and zoonotic transmission [11]. *B. pilosicoli* is an aetiological agent of colitis and occasional spirochaetaemia in humans [12], and a cause of porcine intestinal spirochaetosis (PIS) and avian intestinal spirochaetosis (AIS) [13]. It may also cause disease in other species [8]. *B. pilosicoli* is commonly found in humans living in densely populated areas with poor hygienic conditions [14-17], and in homosexual males [18]. *B. pilosicoli* infections are highly prevalent in intensively farmed swine and poultry, inducing inflammation in the colon and caeca, diarrhoea and reducing growth and productivity [13]. Chemotaxis and motility are deemed important virulence factors, and, as with *B. hyodysenteriae*, *B. pilosicoli* has a chemoattraction to mucin that facilitates penetration of the mucus and association with the underlying intestinal epithelial surface [19,20]. The intimate contact with the epithelia induces a mucus outpouring and epithelial sloughing [21]. An unusual feature of *B. pilosicoli* infection, and shared only by *B. aalborgi*, is the ability to insert one cell end into the luminal surface of enterocytes in the large intestine, forming a pit-like structure, with arrays of such attached spirochaetes giving the appearance of a “false brush-border” [22,23]. This unusual form of attachment of *B. pilosicoli* also occurs in Caco-2 cells *in vitro*, resulting in apoptosis, actin rearrangement and elevated interleukin expression [24].

The paucity of genomic information and absence of tools for genetic manipulation are responsible, at least partly, for the lack of knowledge regarding the adaptations that *Brachyspira* have undergone to colonise the lower GI tract, and for the pathogenic species to induce disease. *Brachyspira* whole genome sequences have only recently been made available for the following species: *B. hyodysenteriae*[25], *B. intermedia*[26], *B. murdochii*[27] and *B. pilosicoli*[28]. The four published sequences showed substantial genetic diversity, and their availability has facilitated research on the corresponding species. However, the availability of only one genome sequence per species has limited the conclusions that can be drawn from the genome as a representation for the species as a whole, and does not allow analysis of intra-species genomic variation. Here, we report the whole genome sequence of *B. pilosicoli* B2904, isolated from a chicken exhibiting clinical symptoms of AIS in the UK, and the partial genome sequence of *B. pilosicoli* WesB, isolated from an Australian Aboriginal child with diarrhoea. Experimentally, the latter strain has been shown to colonise and cause disease in pigs [22]. Although the strains were originally isolated from different host species, it is unlikely that the differences that were found between them were related to their host species of origin [11]. The genomes are presented alongside the whole genome sequence of *B. pilosicoli* 95/1000, isolated from a pig with PIS in Australia, and which has been confirmed to be virulent in experimental infection studies in pigs [22]. We employed the Biolog Phenotype MicroArray™ (PM) technology [29,30] to assess carbon utilisation in these strains. These studies facilitated the validation of differences observed in genotype and permitted detailed correlation between genotype and phenotype.

## Methods

### Bacterial strains and growth conditions

*B. pilosicoli* B2904 was isolated from a chicken displaying clinical symptoms of AIS in the UK; WesB was isolated from an Australian child with diarrhoea [14] and 95/1000 from the diarrhoeic faeces of a pig with PIS, in Australia [22]. The strains were cultured on fastidious anaerobe blood agar (FABA) [31] in an anaerobic atmosphere (10% H_2_ and 10% CO_2_ in N_2_) at 37°C or 42°C for 5 days, for phenotypic studies. For genomic DNA extraction, strains were grown in pre-reduced anaerobic broth [32] at 37°C and a cell pellet was prepared from mid-log phase broth growth.

### Genomic DNA preparation, library construction and sequencing

Cetyltrimethylammonium bromide (CTAB) extraction was used to purify high molecular weight genomic DNA [33]. The *B. pilosicoli* B2904 and WesB genomes were sequenced on a Roche 454 FLX platform, using a standard preparation for a 3 Kb and 8 Kb library, respectively.

For the B2904 genome, a *de novo* assembly of the sequence reads into contiguous sequences was generated using Newbler Assembler software. The reads were assembled into one scaffold of 173 contigs with an average coverage of × 20. Remaining gaps were closed by PCR walking between unlinked, contiguous sequences [33], followed by Sanger sequencing. In total, 170 Sanger reads were incorporated into the assembly.

For the WesB genome, sequence data were initially assembled with Short Oligonucleotide Alignment Program (SOAP) [34] and subsequently Newbler Assembler software was used to create a combined assembly with Illumina reads. Iterative Mapping and Assembly for Gap Elimination (IMAGE) [35] improved genome assemblies by targeted re-assembly of Illumina reads to span gaps within scaffolds. To check for indels (insertion/deletions) and single nucleotide polymorphisms (SNP), Iterative Correction of Reference Nucleotides (iCORN) [36] was applied to the genome and appropriate corrections were made. All repeats over 100 bp were checked to ensure that they were confirmed by at least two spanning read pairs. The incomplete WesB genome was sequenced within one scaffold, with an average coverage of × 34.

### Sequence analysis and annotation

The complete nucleotide sequence and annotation of *B. pilosicoli* B2904 (accession number: CP003490 Project ID: 80999) and partial nucleotide sequence and annotation of *B. pilosicoli* WesB B2904 (accession number HE793032; Project ID: 89437) have been deposited in GenBank. Scaffold sequences for unpublished genomes *B. alvinipulli* C1^T^ and *B. intermedia* HB60 can be accessed from the authors via e-mail request. The draft genome scaffolds for *B. aalborgii* are available at the MetaHit website (http://www.sanger.ac.uk/resources/downloads/bacteria/metahit/).

Sequence and protein analysis and annotation (including rRNA and tRNA prediction) for the complete *B. pilosicoli* B2904 and partial *B. pilosicoli* WesB genomes was as previously described for *B. hyodysenteriae* WA1 [25] and *B. pilosicoli* 95/1000 [28] unless otherwise stated.

The Multi Locus Sequence Typing (MLST) dendrogram of six *Brachyspira* strains that have undergone genome sequencing, and three that are currently within unpublished genome sequencing projects being undertaken by the authors was calculated and constructed from the concatenation of 7 gene nucleotide sequences (*adh*, *pgm*, *est*, *glp*, *gdh*, *thi*, *alp*) [37]. These concatenated sequences were aligned by ClustalW [38] and the maximum likelihood dendrogram was generated via MEGA5 [39]. The condensed bootstrap maximum likelihood dendrogram was constructed from the General Time Reversible (GTR) model with a Gamma of 2.83 (+G) and an assumption that a fraction of sites (0.27) are evolutionarily invariable (+*I*).

The open source utility ‘Freckle’ was used for sequence dot plotting (code.google.com/p/freckle/). Gene prediction and gene and protein sequence extraction was achieved using prodigal 2.50 (prodigal.ornl.gov/).

Initial coding region annotation was completed with an in-house updated compilation of the annotation pipeline AutoFACT 3.4 [40]. Resulting annotations were manually checked and edited where appropriate to be consistent with previous *Brachyspira* genome annotation methodologies for comparative purposes [25,26,28]. Final annotations were assessed with the NCBI Microbial Genome Submission Tool (preview.ncbi.nlm.nih.gov/genomes/frameshifts/frameshifts.cgi).

### Protein cluster analysis

Protein reciprocal blast similarity searches with a threshold maximum expected value 1e-20 were conducted with BlastlineMCL, which is an implementation of the Markov clustering algorithm (MCL) for graphs (http://www.micans.org/mcl/). The granularity of the output cluster was set with an inflation value of 2.5.

### Biolog phenotype MicroArray™

*B. pilosicoli* 95/1000, B2904 and WesB were analysed using the Biolog PM™ technology [29] for high throughput substrate utilisation screening, which included 191 unique carbon sources (PM1 and PM2). PM panels and reagents were supplied by Biolog and used according to the manufacturer’s instructions. Briefly, under anaerobic conditions, bacterial cells were aseptically picked from the FABA agar surface with a sterile cotton swab and suspended in 10 ml of Biolog inoculating fluid (IF-0) until a cell density of 40% transmittance was reached on a Biolog turbidimeter. Before the addition to PM microtitre plates, bacterial suspensions were further diluted into 12 ml of IF-0 (per plate) in sterile water. PM microtitre plates were pre-incubated with two AGELESS® oxygen absorbers (Mitsubishi) 48 h prior to inoculation, at ambient temperature. The resuspended bacterial cells were pipetted into the 96-well plates at a volume of 100 μl/well. Prior to removal from the anaerobic chamber, one AGELESS® oxygen absorber and one CO_2_GEN compact sachet (Oxoid) were attached per PM panel, which were then placed into 4 oz Whirl-Pak® Long-Term Sample Retention Bags (Nasco) with the open end heat-sealed.

Substrate utilisation was measured via the reduction of a tetrazolium dye (clear yellow) to formazan (purple), indicative of cellular respiration at 37°C. Experiments were also run at 42°C, using bacteria cultured at this temperature. Formazan formation was monitored at 15 min intervals for 120 h in OmniLog apparatus. Kinetic data were analyzed with OmniLog-PM software. Each experiment was performed at least twice per strain. It was noted that although tetrazolium dye reduction is indicative of cellular respiration, it can occur independent of cell growth [29,30].

Blank PM1 and PM2 controls were run, whereby IF-0 was added in place of the bacterial cell suspension, to assess for abiotic reactions that occur in the anaerobic atmosphere across the 120 h monitoring period. The following compounds were omitted from analysis due to the nature of the abiotic reactions that occurred in wells containing these compounds, under the conditions of the study: D-arabinose and L-arabinose, dihydroxyacetone, D-glucosamine, 5-keto-D-gluconate, L-lyxose, palatinose, D-ribose, 2-deoxy-D-ribose, sorbate, D-tagatose and D-xylose.

## Results and discussion

### Comparison of general genome features

**Figure 1:**
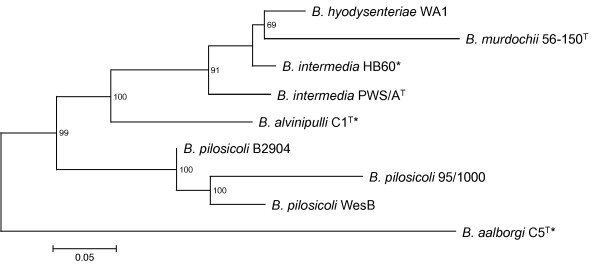
**Dendrogram showing relationships among nine *Brachyspira *strains, representing six of the seven known species.** Analysis was based on concatenated DNA sequences of seven MLST loci [37]. The genome sequences of the strains used in the analysis have either been completed or are currently within a genome sequencing project (*). The tree was constructed using the maximum likelihood method. Bootstrap values (%) are shown for stable nodes. The length of the scale bar is equivalent.

A dendrogram based on the MLST data for nine *Brachyspira* strains highlighted the close relationship between the three *B. pilosicoli* strains, with *B. aalborgi* being distinct, but most closely related to *B. pilosicoli* and distantly related to *B. hyodysenteriae* (Figure 1)*,* concordant with previous findings [26,28]. The two *B. intermedia* strains appeared less closely related than might be expected, supporting reports of extensive diversity in this species based on results of pulse-field gel electrophoresis (PFGE) [41], and a previous MLST study which indicated that these two strains belong to distinct groups [42]. It has been suggested that not all isolates with the *B. intermedia* phenotype should be assigned to this species [26].

**Figure 2:**
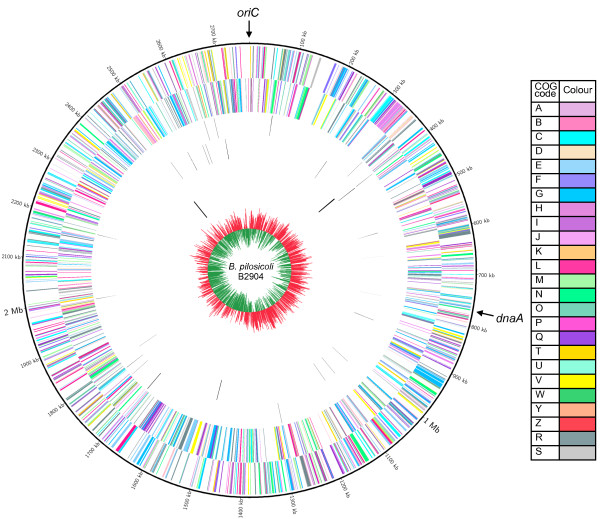
**Circos circular representation of the complete *B. pilosicoli *B2904 genome with annotated genes.** The genome is orientated from the *oriC* and also displays the location of *dnaA*. Circles range from 1 (outer circle) to 7 (inner circle). Circle 1, COG-coded forward strand genes; circle 2, COG-coded reverse strand genes; circle 3, forward strand tRNA; circle 4, reverse strand tRNA; circle 5, forward strand rRNA; circle 6, reverse strand rRNA; circle 7, GC skew ((G-C)/(G + C); red indicates values >0; green indicates values <0). All genes are colour-coded according to Cluster of Orthologous Group (COG) functions shown in the key table; **A**, RNA processing and modification; **B**, chromatin structure and dynamics; **C**, energy production and conversion; **D**, cell cycle control, cell division and chromosome partitioning; **E**, amino acid transport and metabolism; **F**, nucleotide transport and metabolism; **G**, carbohydrate transport and metabolism; **H**, coenzyme transport and metabolism; **I**, lipid transport and metabolism; **J**, translation, ribosomal structure and biogenesis; **K**, transcription; **L**, replication, recombination and repair; **M**, cell wall, membrane and envelope biogenesis; **N**, cell motility and secretion; **O**, posttranslational modification, protein turnover and chaperones; **P**, inorganic ion transport and metabolism; **Q**, secondary metabolite biosynthesis, transport and catabolism; **T**, signal transduction mechanisms; **U**, intracellular trafficking, secretion and vesicular transport; **V**, defence mechanisms; **W**, extracellular structures; **Y**, nuclear structure wheat for cell division and chromosome partitioning; **Z**, cytoskeleton; **R**, general function prediction only; **S**, function unknown.

The general genome features of the three sequenced *B. pilosicoli* genomes are compared in Table 1. The G + C content of the *B. pilosicoli* genomes were very similar to each other (27.44% to 27.9%), and to that of other chromosomes in the genus, which range from 27.1% to 27.9% [26]. The complete genome sequence of *B. pilosicoli* B2904 consisted of a 2,765,477 bp circular chromosome (Figure 2), whereas the incomplete WesB genome was larger, at 2,889,522 bp. The 2,586,443 bp genome of strain 95/1000 was the smallest of the three genomes. Not only did the *B. pilosicoli* genomes show size variability, but also they were smaller than the genomes of the other sequenced species, apart from *B. aalborgi* 513^T^ which our preliminary studies suggest is ~2.5 Mb (unpublished data). The relatively small size of the *B. pilosicoli* genomes is most likely due to them being members of a more specialised species that has undergone a high degree of reductive genome evolution. If this is the case, then *B. pilosicoli* is likely to be an older pathogen than other *Brachyspira* species such as *B. hyodysenteriae*[26]. Such a reductive genome evolution may have allowed improved energy efficiency, and enhanced pathogenic potential. Reductive genome evolution is particularly evident in obligate, intracellular bacterial pathogens [43] and consistent with this, of the *Brachyspira* species, only *B. pilosicoli* and *B. aalborgi* show long-term intimate associations with the surface of enterocytes, into which they interdigitate one of their cell ends. In addition to their small genomes, the sequenced *B. pilosicoli* strains lacked plasmids, whereas the genomes of the other fully sequenced *Brachyspira* species have included plasmids [26].

**Table 1:** **General genome feature comparison for *B. pilosicoli *strains of different host origin**

**Genome features**	**95/1000**	**B2904**	**WesB**^***a***^
Genome size (bp)	2586443	2765477	2889522
G+C content	27.90%	27.79%	27.45%
Total predicted ORFs	2339	2696	2690
Non-significant PID and coverage ORFs	3	23	101
Significant PID and/or coverage ORFs	2336	2673	2589
rRNA genes	3	3	3
tRNA genes	34	34	34
tmRNA genes	1	1	1
hypothetical/conserved hypothetical proteins	657	590	545
genes with function prediction	1641	2045	2006
Genes assigned to COG^*b*^	1201	1196	1276
Genes assigned a KO number^*bc*^	1048	1082	1128
Genes assigned E.C. numbers^*b*^	523	567	563
Genes with signal peptide	244	322	316
Genes with transmembrane helices	48	61	68
Mobile genetic elements (MGE)	4	61	31
insertion sequence elements (ISE)	0	15	17
integrases	0	43	10
transposases	2	1	2
recombinases	2	2	2
Suspected truncated proteins	55	130	64
Suspected protein frameshift/deletions	4	223	50

The comparison includes strains 95/1000 (porcine), B2904 (avian) and WesB (human).

^*a*^ The incomplete WesB strain genome was within one scaffold.

^*b*^ Those genes with significant PID and/or query/target coverage hits; significance equals blastx/blastp PID of at least 25% and/or 75% query or target coverage.

^*c*^ Assigned to KO via KEGG Automatic Annotation Server (KAAS)

The disparity between the number of open reading frames (ORF) and genome size between the B2904 and WesB strains and the high number of non-significant percentage identity (PID) and coverage ORFs in the WesB genome may be an artefact of the incomplete nature of this genome, which is the largest of the three strains. In 95/1000, 44.8% of ORFs were assigned a KEGG Orthology (KO), whereas only 40.5% and 43.6% of ORFs were assigned in B2904 and WesB, respectively. A lower proportion of ORFs were matched in COG database for B2904 and WesB compared to 95/1000.

All three *B. pilosicoli* strains harboured the same number of transfer RNA (tRNA), rRNA and transfer-messenger (tmRNA) genes (Table 1). The tRNA genes represented all 20 amino acids and there were single copies of the 5S, 16S and 23S rRNA genes. The *rrf* (5S) and *rrl* (23S) genes were co-located in all three *B. pilosicoli* genomes, with the *rrs* (16S) gene located approximately 645 Kb, 679 Kb and 773 Kb from the other rRNA genes in the 95/1000, B2904 and WesB genomes, respectively. This rRNA gene organisation has been considered a distinguishing feature of *Brachyspira* species [44], since other spirochaetes typically have differing copy numbers and organisations [45,46]; however, similar arrangements to *Brachyspira* have been detected in the spirochaete *Borrelia burgdorferi*[47]. Situated between the *rrs* gene and *rrfrrl* cluster, which are either side of the *oriC*, was the tmRNA (*ssrA*, 10Sa RNA) gene and nine of the total 34 tRNAs that were otherwise dispersed throughout the genome (Figure 2).

The origin of replication of the *B. pilosicoli* genomes was set according to the position of the *oriC* and GC-skew pattern, as previously suggested [26]; this was supported by the Ori-Finder program [48]. The origin of replication was originally considered to be adjacent to the *dnaA* gene [25,28], however there was no association between the *oriC* and *dnaA* genes in the *B. pilosicoli* B2904 genome (Figure 2), as found in other *Brachyspira* genomes [26]. The arrangement of genes surrounding the *dnaA* gene was consistent between the *B. pilosicoli* strains, as with the other sequenced *Brachyspira* genomes [28]. The genes at the *oriC*, although consistent between the *B. pilosicoli* strains, appear to vary extensively between the species.

### *B. pilosicoli* genome architecture

**Figure 3:**
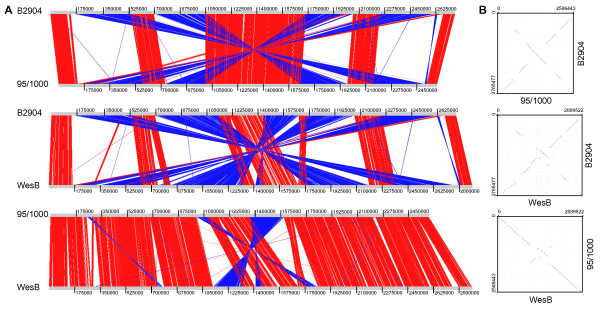
**Pairwise genome alignments and dot matrix plots comparing the genomes of *B. pilosicoli *strains 95/1000, B2904 and WesB.** The Artemis Comparison Tool (ACT) was used to compare the three genome sequences against each other (**A**). Genome sequences were aligned from the predicted *oriC* and visualised in ACT with a cut-off set to blast scores >500. Red and blue bars indicate regions of similarity in the same orientation (red) and inverted (blue). Dot matrix plots of the genome sequences linearised at the o*riC* were generated using Freckle (**B**). The incomplete WesB strain genome was within one scaffold. The output displays a two-dimensional plot, whereby the dots represent matched regions between the genomes. The minimum size of matched sequences was set to 20 bp.

On comparing B2904 with 95/1000, four major genome rearrangement events appeared to have occurred, whereas two rearrangements were evident when comparing WesB to 95/1000 (Figure 3A). Mobile genetic elements (MGE) were found adjacent to or within close proximity of the sites where recombination events appear to have occurred in the B2904 and WesB genomes. Sixty-one and 31 MGEs, including insertion sequence elements (ISE), integrases, recombinases and transposases were identified in the B2904 and WesB genomes, respectively, compared to just four in the 95/1000 genome (Table 1). The proportion of these features therefore seems to correlate with the extent of rearrangement within the genome. Furthermore, multiple copies of an integrase gene that was absent from the 95/1000 genome were identified in the genomes of B2904 (n = 43) and WesB (n = 7) (Additional file 1). The lower number of copies in WesB may be an artefact of the genes not assembling in the incomplete genome. MGEs have been implicated in chromosomal rearrangements, gene disruptions resulting in pseudogenes, and eventual loss of genes, which may contribute to reductive genome evolution [49]. Species and strains that are undergoing or have recently undergone reductive genome evolution, and hence become more specialised pathogens, typically harbour large numbers of MGEs [50-52].

There were fewest suspected pseudogenes (gene truncation or frameshift) found in 95/1000 and most in B2904 (Table 1), a finding that correlates to the number of MGEs and degree of genome rearrangements in these strains. Of the total number of pseudogenes in each strain, 91.5%, 84.5% and 81.3% were in a cluster with orthologs in the other two strains in 95/1000, B2904 and WesB, respectively. Most strikingly, all shared clusters included either multiple B2904 and/or WesB pseudogenes with a complete 95/1000 gene.

Differences in the number of MGEs in the three *B. pilosicoli* genomes may relate to their different stages of reductive genome evolution. Strain 95/1000, which had the smallest genome, also had the fewest MGEs and this could be interpreted as indicating that the MGEs that induced the genome reduction in this strain have become lost. Alternatively, MGE expansion may not have occurred in 95/1000 to the same degree as in B2904 and WesB, as MGEs are generally lost in a fragmentary manner by pseudogenisation. Thus, it is unlikely that the 95/1000 genome, which has the fewest pseudogenes has been reduced in this way. On the other hand, the greater number of pseudogenes in the larger B2904 and WesB genomes does suggest that they may be undergoing genome reduction. A possible explanation would be that these strains are in the initial stages of genome reduction, at the point at which MGE expansion occurs [49,52]. Genome reduction and MGE expansion is often associated with niche specialisation or host restriction [53,54], however *B. pilosicoli* are not considered host-restricted, and WesB, of human origin, has been shown also to infect chickens and pigs [22,55]. *B. pilosicoli* is a highly recombinant species [56], and despite differences in genome arrangement and the number of pseudogenes, part of the difference in genome sizes simply reflects the carriage of different subsets of the pan-genome.

A dot plot comparison of the three *B. pilosicoli* genomes revealed that the chromosomal rearrangements were arranged symmetrically around the origin or terminus of replication, highlighted by the X-patterns in the alignments (Figure 3B). It has been postulated that symmetrical rearrangements occur because recombination events are determined by the replication forks that are approximately equal distance from the o*riC* during bidirectional replication [57]. It has also been argued that non-symmetrical rearrangements can be disadvantageous, and so genome rearrangements such as those found in the *B. pilosicoli* strains are a product of selection [58].

Despite the significant chromosomal rearrangements, genome alignments showed that the majority of genome sequence was shared between the three strains, with the larger B2904 and WesB genomes possessing the greatest proportion of unique sequences (Figure 3). Furthermore, a 26 Kb region, likely to have involvement in horizontal gene transfer (HGT), and that is partially conserved in all previously reported *Brachyspira* genomes as well as *Enterococcus faecalis* and *Escherichia coli*[59], was identified in the *B. pilosicoli* B2904 (B2904_orf2096 – B2904_orf2111) and WesB (wesB2037 – wesB2051) genomes.

### Functional genome comparisons

Functional classifications assigned to each of the protein-coding genes of the three *B. pilosicoli* strains using the COG database showed that the general distribution of features into categories was similar for the three strains (Table 2), and this highlighted their close relationship. Despite having the smallest genome, *B. pilosicoli* 95/1000 possessed the greatest number of features in six categories. B2904 contained the most features in eight categories, and WesB in one category. A striking difference between the strains was in the carbohydrate (G), amino acid (E) and nucleotide (F) transport and metabolism categories, with the larger WesB genome containing considerably more features than the B2904 and 95/1000 genomes. In addition, compared to other *Brachyspira* species the *B. pilosicoli* strains had a reduced number of features associated with inorganic ion transport and metabolism (P) [26,28].

**Table 2:** **Distribution of Cluster of Orthologous Genes (COG) categories in *B. pilosicoli *strains 95/1000, B2904 and WesB**^a^

**Function (COG category)**	**95/1000**	**%**	**B2904**	**%**	**WesB**^***c***^	**%**
**Cellular Processes**						
Translation, ribosomal structure and biogenesis (J)	122	5.22	119	4.45	125	4.83
Transcription (K)	51	2.18	49	1.83	61	2.36
Replication, recombination and repair (L)	51	2.18	56	2.10	61	2.36
**Cellular Processes and Signalling**						
Cell cycle control, cell division and chromosome partitioning (D)	10	0.43	8	0.30	9	0.35
Defence mechanisms (V)	35	1.50	33	1.23	35	1.35
Signal transduction mechanisms (T)	16	0.68	15	0.56	15	0.58
Cell wall, membrane and envelope biogenesis (M)	74	3.17	72	2.69	79	3.05
Cell motility (N)	40	1.71	39	1.46	40	1.54
Intracellular trafficking, secretion and vesicular transport (U)	11	0.47	7	0.26	9	0.35
Posttranslational modification, protein turnover and chaperones (O)	40	1.71	36	1.35	39	1.51
**Metabolism**						
Energy production and conservation (C)	89	3.81	84	3.14	84	3.24
Carbohydrate transport and metabolism (G)	101	4.32	123	4.60	139	5.37
Amino acid transport and metabolism (E)	141	6.03	138	5.16	149	5.76
Nucleotide transport and metabolism (F)	49	2.10	54	2.02	57	2.20
Coenzyme transport and metabolism (H)	47	2.01	44	1.65	48	1.85
Lipid transport and metabolism (I)	41	1.75	33	1.23	34	1.31
Inorganic ion transport and metabolism (P)	53	2.27	53	1.98	49	1.89
Secondary metabolites biosynthesis, transport and catabolism (Q)	9	0.38	10	0.37	9	0.35
**Poorly characterised**						
General function prediction only (R)	149	6.37	147	5.50	157	6.06
Function unknown (S)	72	3.08	76	2.84	77	2.97
**Unassigned**						
Not in COG (X)	1137	48.63	1477	55.26	1313	50.71
**TOTAL**	2338	100	2673	100	2589	100

^*a*^The number and percentage of the total genes within each of the genomes, assigned to each functional group are shown^*b*^.

^*b*^Those genes with significant PID and/or query/target coverage hits; significance equals blastx/blastp PID of at least 25% and/or 75% query or target coverage.

^*c*^ The incomplete WesB strain genome was within one scaffold.

### Global genome feature comparisons

**Figure 4:**
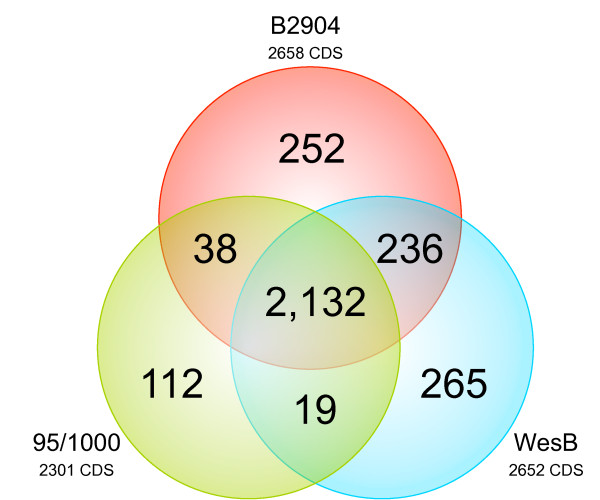
**Venn diagram of genes unique to and shared between *B. pilosicoli *strains 95/1000, B2904 and WesB.** The Venn diagram was resolved via BLASTlineMCL protein clustering. Each circle represents the total number of protein-coding genes in the genome, whereby overlapping regions indicate the number of genes shared between the respective genomes.

The three *B. pilosicoli* strains contained 2,132 conserved genes, and these contribute to defining the *B. pilosicoli* pan-genome (Figure 4); this related to 92.6%, 80.2% and 80.4% of the total genes of the 95/1000, B2904 and WesB genomes, respectively. As expected, there was a greater number of core genes between the strains of *B. pilosicoli* than between strains of different species; substantially fewer core genes (1,087) were identified for *B. hyodysenteriae* WA1, *B. pilosicoli* 95/1000 and *B. murdochii* 56-150^T^[28]. *B. pilosicoli* WesB harboured the greatest number of unique genes, with 10.0% of its genes being absent from the other genomes; B2904 had a similar proportion (9.5%), whereas 95/1000 had considerably fewer (4.9%). *B. pilosicoli* B2904 and WesB shared the greatest proportion of genes (~8.9%) while B2904 shared a greater percentage of its genes with 95/1000 (1.4%) than with WesB (0.7%).

### Global genome feature comparisons against other *Brachyspira* species

Complete genome sequences of *B. hyodysenteriae* WA1, *B. intermedia* PWS/A^T^, *B. pilosicoli* 95/1000 and *B. murdochii* 56-150^T^ have previously undergone comparative analysis [26,28]. Genome sequences of *B. pilosicoli* B2904 and WesB can now be added to these comparisons, giving the first opportunity for a *Brachyspira* intra-species genome comparison. A protein blastmatrix comparison was performed on the four previously sequenced genomes, the two newly-sequenced *B. pilosicoli* genomes and the draft genome scaffolds of *B. aalborgi* 513^T^, *B. alvinipulli* C1^T^ and *B. intermedia* HB60 (unpublished) (Table 3). Not unexpectedly, the analysis revealed that the *B. pilosicoli* strains shared the greatest proportion of proteins (54.9-68.4%). Of *B. pilosicoli* strains, B2904 had the greatest proportion of protein repeats relating to paralogs (2.7%), despite not having the largest genome. Overall, the non-pathogenic *B. murdochii* had the greatest proportion of protein repeats (5.3%), perhaps relating to its large genome. High proportions of shared proteins highlighted the close relationships of *B. hyodysenteriae* with *B. intermedia* (>46.7%) and *B. murdochii* (33.7%) (Figure 1). *B. aalborgi* shared the lowest percentage of proteins with other *Brachyspira* species, consistent with evidence that this is the most divergent species (Figure 1).

**Table 3:** **Protein blastmatrix analysis of nine *Brachyspira *genomes**

	***B. aalborgi***	***B. alvinipulli***	***B. hyodysenteriae***	***B. intermedia***	***B. intermedia***	***B. murdochii***	***B. pilosicoli***	***B. pilosicoli***	***B. pilosicoli***
	**513**^**T*a***^	**C1**^**T*a***^	**WA1**	**PWS/A**^**T**^	**HB60**^***a***^	**56-150**^**T**^	**B2904**	**95/1000**	**WesB**^***b***^
	**2257 CDS**	**3228 CDS**	**2613 CDS**	**2890 CDS**	**3392 CDS**	**2809 CDS**	**2658 CDS**	**2301 CDS**	**2652 CDS**
**WesB**^***b***^	21.36%	21.65%	22.73%	24.95%	19.84%	25.95%	68.43%	65.32%	1.73%
**95/1000**	17.94%	18.56%	20.78%	20.90%	16.77%	22.11%	54.93%	0.74%	
**B2904**	20.47%	21.25%	21.74%	23.60%	19.25%	25.35%	2.71%		
**56-150**^**T**^	22.37%	31.51%	33.68%	36.40%	29.33%	5.30%			
**HB60**^***a***^	20.65%	31.23%	46.77%	57.65%	1.77%				
**PWS/A**^**T**^	21.05%	32.03%	50.33%	1.56%					
**WA1**	17.72%	27.48%	1.11%						
**C1**^**T*a***^	23.48%	2.54%							
**513**^**T*a***^	1.11%								

The percentage of the total CDS that were identified in other genomes (green) and the proportion of protein repeats within the genome (red), is shown. A cut-off e-value of 1e-05 was used.

^*a*^ Incomplete genome currently within a genome sequencing project.

^*b*^ The incomplete WesB strain genome was within one scaffold.

A protein Markov clustering analysis of the six published *Brachyspira* genomes, identified 1,647 protein clusters shared by all six strains (Additional file 2), the encoding genes of which may be used to define a *Brachyspira* species pan-genome. This analysis revealed *B. intermedia* PWS/A^T^ harboured the greatest number of clusters not found in the other sequenced *Brachyspira* genomes (n = 277) and it has the largest genome. The greatest number of clusters shared only between two strains was with *B. intermedia* PWS/A^T^ and *B. hyodysenteriae* WA1 (n = 61), consistent with the close relationship of these species (Figure 1). Of the *B. pilosicoli* strains, B2904 and WesB shared the most unique protein clusters (n = 47), and WesB also shared the greatest number of clusters with a non-*B. pilosicoli* strain, having 36 clusters in common with *B. intermedia* and 16 with *B. murdochii*. The *B. pilosicoli* strains collectively shared the most clusters with *B. murdochii* 56-150^T^ (n = 58), and fewest with *B. hyodysenteriae* WA1 (n = 4), as noted previously [26]. Non-*B. pilosicoli* strains shared 173 clusters, whereas the *B. pilosicoli* strains shared 110 clusters, reflecting gene loss and genome reduction.

#### Unique to the three strains of *B. pilosicoli*

Of 110 protein clusters present only in the *B. pilosicoli* genomes (Additional file 2), 54.6% were hypothetical or unclassified. The majority of protein clusters were metabolic features, including an α-galactosidase (BP951000_0276; B2904_orf1586; wesB_1069), the activity of which is a distinguishing feature of the species [60,61]. Although it was suggested that *B. pilosicoli* had lost many transport-related genes during reductive evolution [26], 13 clusters were for transport proteins. Sialidase family-like protein genes unique to *B. pilosicoli* 95/1000 (BP951000_2021, BP951000_2022 and BP951000_2023) [26,28] were also present in B2904 (B2904_orf1812, B2904_orf1813 and B2904_orf1814) and WesB (wesB_0922, wesB_0923 and wesB_0924); the products of such genes are produced by a variety of mucosal pathogens may play a role in colonisation or inducing tissue damage [62-64]. Clusters for an α-1,2-fucosyl transferase (BP951000_1232; B2904_orf14; wesB_0014), two membrane proteins (BP951000_1751; B2904_orf2268; wesB_0587) (BP951000_1752; B2904_orf2267; wesB_0586) and two glycosyltransferases (BP951000_0003; B2904_orf1276; wesB_1428) (BP951000_2338; B2904_orf1277 and B2904_orf1282; wesB_1429) were unique to *B. pilosicoli* and may contribute to host cell adherence. Other *B. pilosicoli*-specific clusters were for an ankyrin repeat protein (BP951000_0080; B2904_orf1369; wesB_1511), a β-lactamase (BP95100_1338; B2904_orf2576; wesB_0148), two peptidases (BP951000_1129; B2904_orf205; wesB_2479) (BP951000_1260; B2904_orf40; wesB_0047) and phage proteins (BP951000_1211; B2904_orf2686; wesB_2642) (BP951000_1258; B2904_orf39; wesB_0046).

#### Unique and shared by two strains of *B. pilosicoli*

Of the *B. pilosicoli* strains, B2904 and WesB shared most unique clusters (Additional file 2). Fewer clusters were shared with 95/1000, but of twelve clusters unique to 95/1000 and B2904, all but N-acetyl mannosamine-6-phosphate 2-epimerase (BP951000_2135; B2904_orf1689) were hypothetical. Six clusters were unique to 95/1000 and WesB, all lacking a specified function. Of 47 clusters unique to B2904 and WesB, 51.1% were hypothetical; notable clusters shared between these strains were for a further sialidase-like protein (B2904_orf1811; wesB_0925) and a peptidase (B2904_orf863; wesB_1557). The glycine reductase complex locus of 95/1000 (BP951000_1852 – BP951000_1860) and *B. murdochii* 56-150^T^ (Bmur_2720 – Bmur_2728) [28] was identified in B2904 (B2904_orf665 – B2904_orf673) and WesB (wesB_0746 – wesB_0754), but with an additional ATP-binding cassette (ABC)-type glycine betaine transport component in a separate locus (B2904_orf1065; wesB_1632). Moreover, a cluster for a transposase unique to B2904 (n = 47) and WesB (n = 7) was detected. Genes that were shared only by the larger B2904 and WesB genomes and were absent from 95/1000, without apparent detriment, presumably have some specialised function that is not essential for survival. These features may have been lost from 95/1000, as they are not essential, or acquired in B2904 and WesB, perhaps by HGT.

#### Unique to one strain of *B. pilosicoli*

*B. pilosicoli* 95/1000 harboured the fewest and WesB the most unique features (Additional file 2), correlating with their genome size. As discussed above, the 95/1000 strain may have become more specialised, having lost non-essential features through reductive evolution [43]; alternatively, the absence of orthologs in other strains or species may suggest that these features have been acquired via HGT. Of the strain-unique clusters, 77.7%, 65.9% and 68.1% were for hypothetical proteins in 95/1000, B2904 and WesB, respectively. In 95/1000, unique clusters included a sodium/pantothenate symporter and an outer membrane lipoprotein (BP951000_0731) with a potential role in host cell adherence (BP951000_0634). In B2904, unique clusters included putative phage proteins (B2904_orf136, B2904_orf143 and B2904_orf816), additional glycine reductase complex proteins (B2904_orf2051 and B2904_orf2052) and proteins involved in ascorbate metabolism (B2904_orf1019, B2904_orf1020 and B2904_orf1024) and mannitol metabolism (B2904_orf2446 and B2904_orf2447). In WesB, unique features included mannose/sorbose-specific phosphotransferase system (PTS) components (wesB_1270, wesB_1271 and wesB_1272), fructose-specific PTS components (wesB_2317 and wesB_2318) and a D-allose kinase (wesB_1174). Six unique phage-related features and an integrase were identified at two loci in the WesB genome (wesB_0297, wesB_0298, wesB_2528, wesB_2540, wesB_2545, wesB_2550 and wesB_2567). Interestingly, each of the strains harboured unique genes for ankyrin proteins (BP951000_0037; B2904_orf892 and B2904_orf1944; wesB_0903).

### Comparison of potential virulence features

Virulence factor screening in *Brachyspira* genomes was performed as described previously [25,28], but with the analysis encompassing a greater array of genes, particularly in categories relating to adhesion and/or surface proteins and MGEs (Table 4). The greatest number of potential virulence features was in B2904, however additional features may be identified in the WesB genome once it is completed.

**Table 4:** **The number of genes with potential roles in pathogenesis and virulence in the three *B. pilosicoli *genomes**

**Role of putative gene**	**95/1000**	**B2904**	**WesB**^***a***^
Core genes involved in lipopolysaccharide biosynthesis^*b*^	27	30	32
Chemotaxis			
putative methyl-accepting chemotaxis protein	7	7	10
methyl-accepting chemotaxis protein A (*mcpA*)	2	0	2
methyl-accepting chemotaxis protein B (*mcpB*)	8	11	11
chemotaxis protein	15	15	15
Flagella	42	42	42
Adhesion and membrane protein			
lipoprotein	21	31	29
variable surface protein	3	4	4
integral membrane protein	1	1	1
outer membrane protein	25	25	23
periplasmic protein	25	25	28
inner membrane protein	75	83	83
Host tissue degradation			
haemolysis	12	12	12
phospholipase	2	3	2
peptidase	44	48	48
protease	19	19	17
Oxidative stress	7	7	7
Ankyrin-like protein	31	34	35
Phage and other mobile genetic elements	46	109	100
Total	412	506	501

The analysis categorised the genes from the genomes of *B. pilosicoli* 95/1000, B2904 and WesB.

^*a*^ The incomplete WesB strain genome was within one scaffold.

^*b*^ Core lipooligosaccharide (LOS) biosynthesis genes.

#### Lipooligosaccharides

An *rfbBADC* cluster, encoding proteins for nucleotide sugar biosynthesis and with a suggested role in O-antigen assimilation in bacteria such as *Salmonella*[65,66], was identified on the *B. hyodysenteriae* WA1 plasmid [25]. Although lacking this cluster, the three *B. pilosicoli* strains possessed *rfbA* (BP951000_1687; B2904_orf2229; wesB_0523) and *rfbB* (BP951000_1148; B2904_orf2569; wesB_2572), but *rfbC* was noted only in B2904 (n = 1) and WesB (n = 2) (B2904_orf117; wesB_0130 and wesB_0131). Genes inferred to be involved in the biosynthesis of 3,5-dideoxyhexose, an O-antigen component of lipopolysaccharide (LPS) [67], were found adjacent to the *rfbC* gene(s) in B2904 and WesB; both strains contained *rfbF* (B2904_orf115; wesB_0127) and *rfbG* (B2904_orf116; wesB_0128), but *rfbH* was present only in WesB (wesB_0129). The absence of such genes in the pathogenic strain 95/1000 suggests that they have a limited impact on virulence.

#### Chemotaxis and motility

As with 95/1000, the two other *B. pilosicoli* strains possessed fewer chemotaxis genes than *B. hyodysenteriae* and *B. murdochii* (Table 4) [28]. No *mcpC* genes were found in the three *B. pilosicoli* strains, despite their detection in the genomes of the other fully sequenced *Brachyspira* species. The inter-species differences in the number and complement of chemotaxis-related genes may account for differences in their attraction to mucins and affinity to local host niches [19]. No *mcpA* genes were identified in B2904, but two copies were found in the other *B. pilosicoli* strains. The same complement of chemosensory transducer genes was identified in all three strains, as was the previously described cluster of seven such genes [28]. Differences in the number of chemotaxis-related genes between the three strains may translate from differences in genome size. This may denote a redundancy of features that can be lost without apparent detriment to long-term survival. The same flagella genes were shared by all three *B. pilosicoli* strains.

#### Adhesion and membrane proteins

End-on attachment of the spirochaete to the luminal surface of the lower intestinal tract epithelia is characteristic of *B. pilosicoli* and *B. aalborgi* colonisation [2,68], and hence surface-associated proteins or lipoproteins are potential candidates for virulence. All lipoprotein genes in 95/1000 were found in B2904 and WesB, but these strains also had a predicted secreted lipoprotein (B2904_orf1676; wesB_1576) and a lipoprotein carrier protein, LolA (B2904_orf608; wesB_0637), which anchors lipoproteins to the outer membrane [69]. The same complement of genes encoding variable surface proteins found in 95/1000 [28] and the putative integral membrane virulence factor, MviN (B2904_orf469; wesB_2218) were noted in B2904 and WesB. Genes for outer membrane proteins with a potential role in virulence were identified, including BspA antigens, which may bind fibronectin and initiate a serological response [70], OmpA proteins, similar to proteins implicated in *Leptospira* virulence [71], and Tia invasion determinants. Genes encoding TolC were identified in all three *B. pilosicoli* strains, and this protein has been implicated in host invasion, virulence gene expression, and as an outer membrane component of efflux pumps [72-74]. The periplasmic proteins identified were predicted to be primarily associated with other membrane proteins, and constitute ABC transporters with putative roles in virulence [75]. Gene duplications were largely responsible for the greater number of inner membrane virulence factors in B2904 and WesB, but since they were absent for 95/1000 they were unlikely to have significant impact on virulence. WesB harboured two additional genes encoding OppA, which has suggested involvement in spirochaete-host interactions in *Treponema denticola*[76]. Genes encoding P-type ATPase components, such as *cadA* and *zntA*, were noted in the three strains and these have been implicated in the ability of pathogens to sense and adapt to intracellular environments through heavy metal ion regulation [77,78], in addition to Trk potassium transport components, required for invasion and intracellular growth of *Salmonella*[79]. Genes encoding outer, periplasmic and inner membrane proteins that constitute transport systems implicated in bacterial virulence mechanisms were detected, such as polyamine ABC-type transport, which is important for *Streptococcus pneumoniae* pathogenesis [80], TonB-dependant iron transport, which is related to *Shigella dysenteriae* virulence [81], and PTS systems implicated in the virulence of *Mycobacterium tuberculosis* and *E. coli*[82,83]. Genes were found encoding components of the AcrAB-TolC complex, which confers antibiotic resistance and survival in the GI tract [84], a ferrous iron transporter, *feoB*, for iron acquisition, gut colonisation and intracellular survival of multiple enteropathogens [85,86], and a glutamine transporter gene, *glnQ*, which has been implicated in *Streptococcus* adherence and virulence [87]. In the *B. pilosicoli* strains, an *mgl* operon similar to one with a proposed role in virulence expression in *Treponema pallidum*[88] was noted. Multidrug efflux features were found in all three strains, which aside from drug resistance, are attributed with a range of roles in pathogenesis [89]. Genes for the Sec pathway described in 95/1000 [28], with no needle-associated genes were also noted in B2904 and WesB, with an additional *secA*-like gene in WesB (wesB_0869).

#### Host tissue degradation

The complement of haemolysis-related genes was identical between the three strains. Compared to previous analysis, other genes were detected including a haemolysin, previously undetected in 95/1000 (BP951000_1925) and three streptolysin genes, *sagB* (BP951000_0919; B2904_orf445; wesB_2241), *sagC* (BP951000_0918; B2904_orf446; wesB_2240) and *sagD* (BP951000_0917; B2904_orf447; wesB_2239), involved in β-haemolysis and virulence in streptococci [90,91]. A putative phospholipase/carboxylesterase (B2904_orf1218) was found only in B2904. The three strains contained similar numbers of peptidases and proteases, which may participate in local degradation of host tissues, however 95/1000 lacked peptidase E, which had no effect on protein degradation in *Salmonella* Typhimurium [92], and hence this non-essential enzyme may have been lost through reductive evolution.

#### Oxidative stress

Genes related to oxidative stress were shared by the three strains. A partial *BatI* (*Bacteroides* aerotolerance) operon [93] was noted in all strains, in close proximity to one of the *nox* genes and consisted of *batB* (BP951000_0196; B2904_orf1493; wesB_1155), *batC* (BP951000_0195; B2904_orf1492; wesB_1156), *batD* (BP951000_0194; B2904_orf1491; wesB_1157) and *batE* (BP951000_0193; B2904_orf1490; wesB_1158). The *batA* gene was in a distinct locus in the three strains (BP951000_1387; B2904_orf2546; wesB_0200).

#### Ankyrin-like protein

There was little difference in the number of genes encoding ankyrin-like proteins between the *B. pilosicoli* strains, which may be involved in host cell interactions through their ability to bind host chromatin as in *Orientia*[94]. *B. pilosicoli* had consistently fewer of these genes than *B. hyodysenteriae*[28].

#### Phage and other mobile genetic elements

**Figure 5:**
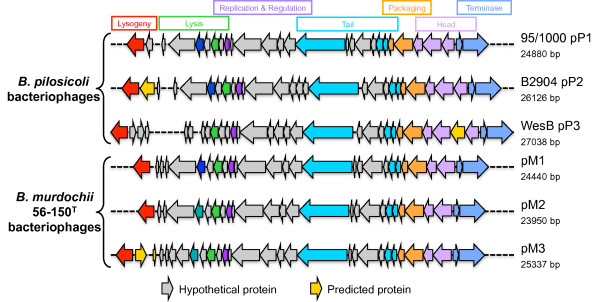
**Comparison of the organisation of the bacteriophages in the three *B. pilosicoli *genomes.** A comparison on bacteriophages pP1 in 95/1000, pP2 in B2904 and pP3 in WesB and also the three bacteriophages found in *B. murdochii* 56-150^T^; pM1, pM2 and pM3. Genes encoding hypothetical proteins (grey) and genes with predicted protein function (yellow) are indicated.

Outside of bacteriophage regions, four, 61 and 31 MGEs were identified in 95/1000, B2904 and WesB, respectively, correlating with the extent of genomic rearrangements. The types and copy number of all MGEs in the *B. pilosicoli* genomes are detailed in Additional file 1. The region encoding genes related to the VSH-1 prophage-like gene transfer agent (GTA) in 95/1000 [28], was identified in B2904 (B2904_orf2669 – B2904_orf2692) and WesB (wesB_2625 – wesB_2648). This region was ~15 Kb in 95/1000 compared to ~21 Kb in B2904 and WesB due to an insertion between genes encoding OrfE and Hvp53, containing genes for a monosaccharide-transporting ATPase (B2904_orf2671; wesB_2628), an ABC transporter-related protein (B2904_orf2672; wesB_2629), a ROK family protein (B2904_orf2674; wesB_2631), an integrase in B2904 only (B2904_orf2675), and a periplasmic binding protein/LacI transcriptional regulator (B2904_orf2673; wesB_2627 and wesB_2630). Generally, these features had high homology with those in *Clostridium carboxidivorans* (e-value < 1e-74), consistent with the finding that *Brachyspira* share a high degree of gene similarity with *Clostridium*[25], and supporting the notion that the bacteriophages exchange genetic material between species [26]. In WesB, an additional cluster of VSH-1-associated genes, flanked by a phage terminase, was detected (wesB_2527 – wesB_2553); the different genes in this region shared highest homology with *C. carboxidivorans*, *B. hyodysenteriae*, *B. intermedia*, *B. pilosicoli* and *B. murdochii*, suggesting that the GTA had involvement in intra- and inter-species gene transfer. The bacteriophage that was identified in *B. pilosicoli* 95/1000 (pP1), and in *B. murdochii* 56-150^T^ (pM1, pM2 and pM3) [26,28], was also found in B2904 (pP2; B2904_orf1942 – B2904_orf1970) and WesB (pP3; wesB_0739 – wesB_0708) (Figure 5). In *B. pilosicoli*, the bacteriophage size was proportional to genome size. Hypothetical proteins encoded in this region were shared between 95/1000 and B2904, however WesB contained four unique hypothetical genes. The B2904 pP2 bacteriophage possessed a unique ankyrin repeat protein (B2904_orf1943). An adenine-specific DNA methyltransferase gene was present only in the WesB pP3 bacteriophage (wesB_0711), adjacent to the DNA methylase gene found in *B. pilosicoli* bacteriophages (BP951000_1480; B2904_orf1968; wesB_0710), but absent from those of *B. murdochii* 56-150^T^. Two separate novel bacteriophages regions were found in B2904 (pP4) and WesB (pP5). The ~29 Kb pP4 bacteriophage contained seven phage proteins (B2904_orf133 – B2904_orf180), six predicted proteins with homology to sequences of other *Brachyspira* species, and 35 unique hypothetical genes. The ~28 Kb pP5 bacteriophage (wesB_0301 – wesB_0341) shared all the components of the pI1 bacteriophage of *B. intermedia* PWS/A^T^, suggesting transfer of the bacteriophage in an inter-species HGT event. Interestingly, pP5 was flanked by VSH-1 components (wesB_0297, wesB_0298 and wesB_0343), and hence the VSH-1 GTA may be responsible for mediating the HGT event. Two nuclease genes (wesB_0306 and wesB_0308) and a number of unique hypothetical genes in pP5 were not identified in pI1. Clustered regularly interspaced short palindromic repeats (CRISPR), which provide bacteria with acquired resistance to bacteriophages [95], were only identified in the non-pathogenic *B. murdochii* 56-150^T^, which suggests a role for bacteriophages in *Brachyspira* pathogenicity. *B. pilosicoli* B2904 and *B. intermedia* PWS/A^T^ did however possess a bacteriophage resistance protein (B2904_orf2624; Bint_2390) which has been implicated in protecting against bacteriophages [96].

### Central metabolism and correlation with phenotype

Analysis of the genomes of *B. hyodysenteriae* and *B. pilosicoli* has revealed that these species share many metabolic capabilities [25,28]. In the current study the analysis of central metabolic pathway detection in *Brachyspira* genomes was extended by application of Biolog PM™ technology for phenotypic determination of carbon source utilisation of the *B. pilosicoli* strains. The utilisation of 178 unique carbon compounds (Additional file 3) by the strains was screened, and their metabolic capabilities were found to be highly conserved. Differences were found in the utilisation of just seven carbon sources, which were correlated with genotypic variations (Table 5).

**Table 5:** **Correlation between differences in carbon source utilisation and genotype of *B. pilosicoli *95/1000, B2904 and WesB**

**Unique carbon source compound tested**	**95/1000**	**B2904**	**WesB**	**Possible explanation**
D-Mannose	-	-	+	WesB is the only strain with the mannose/sorbose-specific PTS system IIABCD components (wesB_1269, wesB_1270, wesB_1271 and wesB_1272) for uptake and phosphorylation of D-mannose.
D-Glucuronic acid	-	+	+	95/1000 lacks the pfkB carbohydrate kinase, 2-dehydro-3-deoxygluconate kinase, which links D-glucuronic acid metabolism to glycolysis. This enzyme is found in both B2904 (B2904_orf899 and B2904_orf900) and WesB (wesB_1781).
D-Mannitol	-	+	-	B2904 is the only strain with the D-mannitol PTS system IIABC components (B2904_orf2447) and also a mannitol-1-phosphate 5-dehydrogenase (B2904_orf2446) for D-mannitol, uptake, phosphorylation and catabolism.
Glucuronamide	-	+	+	95/1000 lacks the pfkB carbohydrate kinase, 2-dehydro-3-deoxygluconate kinase, which links glucuronate and related compound metabolism to glycolysis. This enzyme is found in both B2904 (B2904_orf899 and B2904_orf899 and B2904_orf900) and WesB (wesB_1781).
β-D-Allose	-	-	+	WesB is the only strains with D-allose ABC transporter components (wesB_1171, wesB_1172 and wesB_1175) and D-allose kinase (wesB_0259 and wesB_1174) for uptake and metabolism of D-allose.
β-Methyl-D-glucuronic acid	-	+	+	95/1000 lacks the pfkB carbohydrate kinase, 2-dehydro-3-deoxygluconate kinase, which links glucuronate and related compound metabolism to glycolysis. This enzyme is found in both B2904 (B2904_orf899 and B2904_orf900) and WesB (wesB_1781).
L-Sorbose	-	-	+	WesB is the only strain with the mannose/sorbose-specific PTS system IIABCD (wesB_1269, wesB_1270, wesB_1271, wesB_1272) components for uptake and phosphorylation of L-sorbose.

Possible explanations for the differences in phenotype relate to differences in genomic features.

#### Carbohydrate metabolism

High proportions of the *B. pilosicoli* genomes were associated with carbohydrate transport and metabolism (Table 2), and from metabolic pathway reconstructions it is evident that glycolysis constitutes a major backbone of energy production [28]. Collectively the *B. pilosicoli* strains utilised 51.9% of carbohydrate compounds tested, and more specifically 69.4% of hexose sugars (Additional file 3). Genes for enzymes involved in converting glucose-6-phosphate to ribulose-5-phosphate that were identified in *B. hyodysenteriae* WA1 [25], were found in the *B. pilosicoli* genomes. These features are likely to direct carbohydrate oxidation towards the non-oxidative pentose phosphate pathway, to generate reducing power required for biosynthetic pathways. *B. pilosicoli* is characterised by an absence of β-glucosidase activity [60], however a novel system for metabolising β-glucosides found in 95/1000 [28] was also present in B2904 and WesB, which, alongside specific PTS systems, is likely to be involved in the utilisation of D-cellobiose and arbutin as carbon sources. Despite lacking β-glucosidase, metabolism of β-glucosides may be important to *B. pilosicoli* virulence as this phenotype is associated with growth, adhesion and colonisation in other bacteria [97]. Of the disaccharides tested, 64.3% were utilised by the *B. pilosicoli* strains, whereas, of the oligosaccharides only dextrin was utilised, which is likely to be attributed to α-glucosidase activity (BP951000_1130; B2904_orf204; wesB_2480).

#### Amino acid metabolism

Of the COG categories related to metabolism, the greatest proportion of the genome was related to amino acid transport and metabolism (Table 2). Phenotypic studies revealed that despite the high number of genes for amino acid/ oligopeptide transporters found in the genomes, only five of the tested amino acids were able to support *B. pilosicoli* as a sole carbon source (Additional file 3). Genes encoding enzymes to direct these amino acids towards pyruvate metabolism and hence energy production were identified, including alanine dehydrogenase (BP951000_0036; B2904_orf1321; wesB_1465), threonine aldolase (BP951000_1568; B2904_orf2409; wesB_0396), glycine hydroxymethyltransferase (BP951000_1528; B2904_orf2450; wesB_0361) and L-serine dehydratase (BP951000_0452 and BP951000_0453; B2904_orf939 and B2904_orf940; wesB_1746 and wesB_1747). Moreover, a glycine reductase complex found in the *B. pilosicoli* strains, which catalyses the reductive deamination of glycine, forming ATP, would be involved in the utilisation of glycine. A high proportion of amino acid metabolic features in *B. pilosicoli* were related to biosynthesis and potentially maintaining intermediates of the partial tricarboxylic acid (TCA) cycle identified in this species [28], rather than catabolism to produce energy. L-glutamate and L-glutamine were insufficient to sustain *B. pilosicoli* as a sole carbon source; these amino acids are primary products of ammonia assimilation used in peptidoglycan, LOS and outer membrane protein biosynthesis [98], hence their metabolism is redirected to energy yielding pathways. The *B. pilosicoli* strains possessed genes for glutamate dehydrogenase (BP951000_1312; B2904_orf93; wesB_0103), which catalyses the reversible synthesis of glutamate from α-ketoglutarate and ammonium. Since α-ketoglutarate was able to sustain *B. pilosicoli*, the presence of a transporter for α-ketoglutarate and not glutamate may explain this phenotype. The ability to utilise certain amino acids as an energy source may have become redundant in *Brachyspira*, which typically occupy the nutrient-rich lower GI tract, and thus associated features may have been lost through reductive evolution.

#### Nucleotide metabolism

The *B. pilosicoli* strains were able to utilise three purine and two pyrimidine nucleosides tested as a sole carbon source (Additional file 3). The enzymes suggested to complete a metabolic link between nucleoside and central metabolism in *B. hyodysenteriae* WA1 [25] were identified in the *B. pilosicoli* strains.

#### Lipid metabolism

Despite the presence of enzymes involved in the β-oxidation of fatty acids, including a long chain fatty acid-CoA ligase (BP951000_0887; B2904_orf479; wesB_2210), no long chain fatty acids tested were utilised by *B. pilosicoli* as a carbon source; however, the short chain fatty acids, butyric acid and propionic acid, were utilised (Additional file 3). Glycerol was utilised as a carbon source, and genes for its metabolism were detected including those for a glycerol uptake facilitator (BP951000_0799; B2904_orf2190; wesB_2118), glycerol kinase (BP951000_0800; B2904_orf2191; wesB_2119) and glycerol-3-phosphate dehydrogenase (BP951000_1696; B2904_orf2220; wesB_0532). The gene set required for fatty acid biosynthesis was incomplete in B2904 and WesB, as it was in 95/1000 [28].

## Conclusions

In this study, we report the genome of *B. pilosicoli* strain B2904 and the near complete genome of strain WesB. Together with the previously reported 95/1000 genome, this allowed the first intra-species genome comparison within the genus *Brachyspira*. Our feature-based analysis revealed a high level of similarity between the three strains and identified genes that we suggest different strains of the spirochaete may have lost in a process of reductive genome evolution. Sequence-based comparisons showed the majority of sequence was shared between the strains, with few unique regions; however, genome rearrangements were observed around the *oriC*. MGEs were found associated to areas of rearrangements, and these features may be a factor that has driven or is driving reductive evolution. Novel bacteriophages were identified in the newly-sequenced genomes, which displayed evidence of intra- and inter-species HGT, and these may have key practical applications for use in genetic manipulation. This is the first analysis of the spirochaete in a high-throughput phenotype screening tool, allowing correlation between genotype and phenotype. Future work will focus on the application of this technology to a wider range of *Brachyspira* species to validate genome differences, potentially providing a means by which these phenotypes can be used for rapid screening to infer genotypes and improve current diagnostic methods. With the increasing availability of *Brachyspira* genome sequences, such technology should facilitate the validation of metabolic models based on genome sequence.

## Abbreviations

A: Adenine; AIS: Avian intestinal spirochaetosis; ABC: ATP-binding cassette; ACT: Artemis comparison tool; ATP: Adenosine triphosphate; bp: Base pair; C: Cytosine; CDS: Coding DNA sequence; COG: Cluster of Orthologous Genes; CRISPR: Clustered regularly interspaced short palindromic repeats; CTAB: Cetyltrimethylammonium bromide; DNA: Deoxyribonucleic acid; FABA: Fastidious anaerobe blood agar; G: Guanine; GI: Gastrointestinal; GTA: Gene transfer agent; GTR: General time reversible; HGT: Horizontal gene transfer; iCORN: Iterative correction of reference nucleotides; IMAGE: Iterative mapping and assembly for gap elimination; Indels: Insertions/deletions; ISE: Insertion sequence element; KAAS: KEGG automatic annotation server; Kb: Kilobase; KEGG: Kyoto encyclopedia of genes and genomes; KO: KEGG orthology; LOS: Lipooligosaccharide; LPS: Lipopolysaccharide; Mb: Megabase; MCL: Markov clustering algorithm; MGE: Mobile genetic element; MLST: Multi locus sequence typing; NCBI: National center for biotechnology information; ORF: Open reading frame; PCR: Polymerase chain reaction; PFGE: Pulse-field gel electrophoresis; PID: Percentage identity; PIS: Porcine intestinal spirochaetosis; PM: Phenotype MicroArray^TM^; PTS: Phosphotransferase system; RNA: Ribonucleic acid; rRNA: Ribosomal RNA; SNP: Single nucleotide polymorphisms; SOAP: Short oligonucleotide alignment program; T: Thymine; TCA: Tricarboxylic acid; tmRNA: Transfer-messenger RNA; tRNA: Transfer RNA.

## Competing interests

The authors declare that they have no competing interests.

## Authors’ contributions

LJM, DJH and RML conceived the study. LJM and ACD performed the genome sequencing and gap closure of *B. pilosicoli* B2904. JP and AKT were involved in genome sequencing of the WesB. DJH, TL and NDP were involved in the genome sequencing of *B. aalborgi* 513^T^, *B. alvinipulli* C1^T^ and *B. intermedia* HB60. LJM, MLB, DJH, MIB and ACD conceived and designed comparative genomic studies. LJM and MLB performed comparative genomic analysis and analysed the data. LJM, MA, MJW and RML conceived and designed phenotyping experiments. LJM and MA conducted phenotyping experiments. LJM wrote the manuscript. All authors read and approved the final manuscript.

## Supplementary Material

Additional file 1 **Type and copy number of mobile genetic elements (MGE) in the genomes of*B. pilosicoli *95/1000, B2904 and WesB.** A combination of protein markov cluster analysis and reciprocal blast searches against the conserved domain database (CDD) was used to determine the copy number of each type of MGE across the three *B. pilosicoli* genomes, using a cut-off e-value of 1e-20. The ORF number, position, and size of all MGEs identified in each of the *B. pilosicoli* genomes is displayed. Click here for file

Additional file 2 **Conserved and shared protein clusters between the six genome-sequenced*Brachyspira *strains. ***B. hyodysenteriae* WA1 (H), *B. intermedia* PWS/A^T^ (I), *B. murdochii* 56-150^T^ (M) and *B. pilosicoli* 95/1000 (Pa), B2904 (Pb) and WesB (Pc)^*a*^ strains we included in the protein cluster analysis. A cut-off e-value of 1e-20 was used. Click here for file

Additional file 3 **Comparison of the utilisation of unique carbon sources by*B. pilosicoli*95/1000, B2904 and WesB.** Biolog Phenotype MicroArray™ (PM) technology was employed for these studies and OmniLog apparatus was used to detect formazan formation and hence, respiration due to utilisation of the carbon source; +, able to utilise the compound; -, unable to utilise the compound. Click here for file
